# Development and validation of a 1-year survival prognosis estimation model for Amyotrophic Lateral Sclerosis using manifold learning algorithm UMAP

**DOI:** 10.1038/s41598-020-70125-8

**Published:** 2020-08-07

**Authors:** Vincent Grollemund, Gaétan Le Chat, Marie-Sonia Secchi-Buhour, François Delbot, Jean-François Pradat-Peyre, Peter Bede, Pierre-François Pradat

**Affiliations:** 1 Laboratoire d’Informatique de Paris 6, Sorbonne Université, Paris, 75005 France; 2 FRS Consulting, Paris, 75009 France; 3 Nanterre Université, Modal’X, Nanterre, 92014 France; 4 Laboratoire d’Imagerie Biomédicale, Sorbonne Université, Paris, 75005 France; 5 Département de Neurologie, Pitié-Salpêtrière University Hospital, APHP, Paris, 75013 France; 6 Computational Neuroimaging Group, Trinity College, Dublin, D02 PN40 Ireland; 7 Antnagelvin Hospital, Northern Ireland Center for Stratified Medecine, Biomedical Sciences Research Institute Ulster University, C-TRIC, Londonderry, BT47 6SB United Kingdom

**Keywords:** Neurological disorders, Computer science

## Abstract

Amyotrophic Lateral Sclerosis (ALS) is an inexorably progressive neurodegenerative condition with no effective disease modifying therapies. The development and validation of reliable prognostic models is a recognised research priority. We present a prognostic model for survival in ALS where result uncertainty is taken into account. Patient data were reduced and projected onto a 2D space using Uniform Manifold Approximation and Projection (UMAP), a novel non-linear dimension reduction technique. Information from 5,220 patients was included as development data originating from past clinical trials, and real-world population data as validation data. Predictors included age, gender, region of onset, symptom duration, weight at baseline, functional impairment, and estimated rate of functional loss. UMAP projection of patients shows an informative 2D data distribution. As limited data availability precluded complex model designs, the projection was divided into three zones with relevant survival rates. These rates were defined using confidence bounds: high, intermediate, and low 1-year survival rates at respectively $90\%$ ($\pm 4\%$), $80\%$ ($\pm 4\%$) and $58\%$ ($\pm 4\%$). Predicted 1-year survival was estimated using zone membership. This approach requires a limited set of features, is easily updated, improves with additional patient data, and accounts for results uncertainty.

## Introduction

Amyotrophic Lateral Sclerosis (ALS) is a relentlessly progressive neurodegenerative condition involving both upper and lower motor neurons, leading to progressive limb weakness and bulbar dysfunction. Mean survival time from disease onset is typically 3 to 5 years^1^, with death occurring secondary to respiratory failure. The disease is characterised by considerable clinical heterogeneity^2^ and differences in progression rate^3^, with some patients surviving 10 years or more^4,5^.

From a clinical perspective, accurate prognostic indicators are indispensable for optimising multidisciplinary care, planning interventions, advising patients on end-of-life decisions, resource allocation, etc. Disease heterogeneity is a recognised barrier to successful clinical trials in ALS^6^, and accurate prognosis prediction would improve patient stratification. Previous epidemiology studies have identified a number of negative prognostic indicators^7^, such as older age of onset, bulbar onset, respiratory compromise, cognitive impairment, short symptom onset to diagnosis interval, marked functional disability, c9orf72 status, and fast progression rate^8–11^. However, individualised prediction is seldom reliable when clinical and demographic variables are considered alone^11^. There is a growing trend to develop accurate prognostic tools based a combination of prognostic factors^12^, using supervised machine learning models such as random forests^13^, regression models^14^, neural networks with random forests^15^ and boosting algorithms^16^. Recently, Westeneng et al.^17^ presented an externally validated Royston-Parmar regression prediction model of survival in a large European ALS population.

Unsupervised learning methods provide new modelling opportunities in ALS due to their ability to detect data distributions without a firm underlying statistical hypothesis^18,19^. Dimension reduction methods project data onto a new low-dimensional space and allow interesting data visualisation. A neighbourhood-based approach takes full advantage of patient similarity for prognosis modelling and can unravel relevant correlations between predictors and outcomes. Uniform Manifold Approximation and Projection (UMAP)^20^ is a novel method based on non-linear dimension reduction which can be readily combined with probability assessments. The main objective of our study was to evaluate a UMAP based 1-year survival prediction model in ALS, designed using three clinical trial datasets, and validated by a Real-World (RW) dataset. Model performance was compared with random forest and logistic regression models. The model is easily updated, works with a limited set of features and factors result uncertainty in. Taking advantage of the UMAP projection, other prognosis outcomes and different time frames can be explored.

## Methods

### Patient population

Validation and test data for this research included a total of 5,393 patients from four different datasets, three of which originated from clinical trials. The first dataset, which is referred to as ‘Trophos’, was a clinical trial for olesoxime, a drug developed by Trophos^21^ which included 512 patients. After excluding samples with missing data, 431 patients remained. The second dataset, ‘Exonhit’, was a clinical trial for pentoxifylline, a drug produced by Exonhit Pharma^22^ which included 400 patients. Given the considerable negative effect of the tested treatment on survival time, patients that received the treatment were excluded from outcome analysis. Nevertheless, these patients were included in dimension reduction as projection calculation is solely based on baseline features. Following the exclusion of incomplete samples and patients having received the treatment, data from 345 patients were included in the dimension reduction phase and 172 patients were retained for 1-year survival analysis. The third database was ‘PRO-ACT’, funded by the ALS Therapy Alliance and released in 2012 as part of the DREAM Phil Bowen ALS prediction Prize4Life competition. PRO-ACT consists of pooled data from 16 completed phase II-III clinical trials and one observational study^23^. The original sample size was 10,723, reduced to 3,971 after discarding samples with missing data. The fourth dataset was population-based and contained RW patient data. These data were obtained from the database of the Paris tertiary referral centre for ALS collected between September 1999 and April 2008. The original sample size was 1,377 which was reduced to 646 after the removal of incomplete samples. Baseline patient feature distribution for 1-year survival analysis is presented for each cohort in Table 1. Additional information on each dataset is provided as supplementary information.

**Table 1:** Predictor distribution per dataset.

Source	n	Gender (male/female)	Onset (spinal/bulbar)	Age (years)	Symptom duration (months)	Baseline weight (kg)	Baseline ALSFRS (score)	Baseline ALSFRS decline rate (score/month)
PRO-ACT	3,971	2,485/1,486	3,117/854	$56.2 \pm 11.3$ (18:81)	$20.8 \pm 12.7$ (0.5:140.4)	$74.8 \pm 15.8$ (30:148.6)	$30.1 \pm 5.7$ (7:40)	$-0.61 \pm 0.51$ ($-$6.09:0)
Trophos	431	277/154	346/85	$56.7 \pm 11.1$ (26:79)	$16.4 \pm 8.0$ (5:38)	$71.5 \pm 12.7$ (41:130)	$32.5 \pm 4.1$ (16:40)	$-0.55 \pm 0.39$ ($-$2.67:0)
Exonhit	172	118/54	129/43	$55.6 \pm 12.0$ (26.3:77.9)	$24.7 \pm 11.9$ (5:58)	$70.1 \pm 13.8$ (45:112)	$27.5 \pm 6.4$ (10:39)	$-0.60 \pm 0.40$ ($-$3.14:$-$0.05)
Real world	646	345/301	458/188	$62.2 \pm 12.1$ (26.3:92.2)	$22.1 \pm 21.6$ (0:228.5)	$70.4 \pm 13.2$ (40:140)	$28.6 \pm 7.4$ (3.5:40)	$-0.78 \pm 0.65$ ($-$4.16:0)
Overall	5,220	3,225/1,995	4,050/1,170	$57.0 \pm 11.6$ (18:92.2)	$20.7 \pm 13.9$ (0:228.5)	$73.8 \pm 15.3$ (30:148.6)	$30 \pm 5.9$ (3.5:40)	$-0.63 \pm 0.52$ ($-$6.09:0)

Numerical predictors are described using mean ± standard deviation (range).

#### Clinical predictors and outcomes

The primary outcome was 1-year survival. Overall survival (in months), and 1-year functional loss (using the validated ALS Functional Rating Scale (ALSFRS)) were secondary outcomes. Each outcome had a specific data scope: 1-year survival was a binary variable and was predicted for patients dying within 12 months or with an available ALSFRS score at t+12 months. 1-year functional loss was predicted for patients that survived at t+12 months with an ALSFRS score at that time. Patients who died or had invasive ventilation within the first year were assigned an ALSFRS score of 0 at year 1. Overall survival (in months) was used for patients when such information was available but provides a limited understanding of true patient survival given patient monitoring ended at t+12 months for most data.

The choice of predictors was based on feature completeness after database cross-referencing. Predictors include gender, region of onset (spinal/bulbar), age, symptom duration, baseline ALSFRS score, baseline weight, and estimated functional decline rate^24^. The functional decline rate was estimated using the following formula:

1 $$\begin{aligned} decline\;rate = \frac{ALSFRS_{maximum}-ALSFRS_{baseline}}{symptom\; duration} \end{aligned}$$

with $ALSFRS_{baseline}$, the ALSFRS score recorded at baseline, $ALSFRS_{maximum}$, the maximum score for the ALSFRS (40) and $symptom\; duration$ , time in months between symptom onset and baseline.

Table 2 provides an overview of patient outcome feature distribution. Patient survival was on average above $75\%$ for all datasets, and 1-year average ALSFRS was above 17 for all datasets. Overall patient survival was bounded by clinical trial follow up time.

**Table 2:** Outcome distribution per dataset.

Source	n (1-year survival)	Survival rate (%)	n (survival)	Survival (months)	n (1-year ALSFRS)	1-year ALSFRS (score)
PRO-ACT	3,971	76	1,434	$10 \pm 5$ (0:31)	3,789	$17 \pm 12$ (0:40)
Trophos	431	84	99	$11 \pm 4$ (3:15)	428	$21 \pm 11$ (0:38)
Exonhit	172	72	79	$10 \pm 5$ (1:18)	165	$16 \pm 12$ (0:39)
Real world	646	67	447	$14 \pm 9$ (0:41)	543	$14 \pm 13$ (0:40)
Overall	5,220	75	2,059	$11 \pm 6$ (0:41)	4,925	$17 \pm 12$ (0:40)

Numerical predictors are described using mean ± standard deviation (range).

### Missing data management

Missing feature analysis focused solely on baseline predictors and outcomes (overall survival, 1-year survival, and 1-year ALSFRS). Table 3 presents missing data ratio per feature for all datasets. Features which were not available in all datasets, such as testing and biological lab results, were discarded. ALSFRS sub-scores were not recorded for Trophos patients and were discarded as a whole. Outcome features can easily be missing due to loss to follow up or death. Features at time t+3 were less available than at baseline. Data collection was not disclosed for PRO-ACT data which aggregates multiple clinical trials and this prevented the identification of missing data patterns. Due to data collection differences between the cohorts, we did not perform missing data imputation and opted for complete case analysis.

**Table 3:** Missing feature analysis per dataset.

Group	n	Survived (yes/no)	Gender (male/female)	Onset (spinal/bulbar)	Age (years)	Symptom duration (months)	Baseline weight (kg)	Baseline ALSFRS (score)	Baseline ALSFRS decline rate (score/month)	1-year ALSFRS (score)
High survival rate zone	1,525	1,378/147	1,189/336	1,187/338	$54.1 \pm 9.7$ (22:78)	$16.7 \pm 9.6$ (2.9:59.8)	$82.5 \pm 15.1$ (46:148.6)	$35.4 \pm 2.2$ (27:40)	$-0.34 \pm 0.22$ ($-$1.46:0)	$25.1 \pm 10.6$ (0:40)
Intermediate survival rate zone	1,524	1,219/305	899/625	1,171/353	$56.4 \pm 12.1$ (18:81)	$21.2 \pm 12.7$ (3.1:140.4)	$70.8 \pm 12.5$ (30:122.5)	$31.3 \pm 2.2$ (25:39)	$-0.56 \pm 0.34$ ($-$2.38:$-$0.02)	$18.3 \pm 11.1$ (0:39)
Low survival rate zone	1,525	892/633	792/733	1,234/291	$58.3 \pm 11.6$ (25:80)	$23.7 \pm 13.5$ (0.5:86.7)	$69.6 \pm 15.3$ (36.5:138.9)	$23.9 \pm 4.2$ (7:35)	$-0.92 \pm 0.63$ ($-$6.09:$-$0.15)	$9.0 \pm 9.4$ (0:37)
Overall	4,574	3,489/1,085	2,880/1,694	3,592/982	$56.3 \pm 11.3$ (18:81)	$20.5 \pm 12.4$ (0.5:140.4)	$74.3 \pm 15.5$ (30:148.6)	$30.2 \pm 5.6$ (7:40)	$-0.61 \pm 0.49$ ($-$6.09:0)	$17.6 \pm 12.3$ (0:40)

Numerical predictors are described using mean ± standard deviation (range).

### Data processing

Pre-processing was limited to predictor normalisation to the 0–1 range. Data transformation was carried out through non-linear dimension reduction, also called manifold learning. The Uniform Manifold Approximation and Projection for Dimension Reduction (UMAP)^20^ method was implemented. UMAP works in two steps. First, a compressed embedding of the input space (aka initial patient data) is generated through topological analysis of the data structure. Subsequently, a low-dimensional (in our case 2D) data embedding is created through a cross-entropy optimisation process. UMAP preserves data neighbourhoods, distances and density. ‘Development data’ were used to learn a 2D representation of patients. Validation data were projected using the learnt mapping. Information on the subject can be found in the supplementary information section. Sample size of development data for 1-year survival was 4,574. Functional loss and overall survival analyses had lower sample sizes: respectively 4,382, a $4\%$ drop with regards to 1-year survival sample size, and 1,612, a $65\%$ drop with regards to 1-year survival. Sample size of validation data for 1-year survival, functional loss and overall survival were respectively 646, 541 and 447.

1-year survival rates zones were identified by dividing the UMAP projection space into multiple small square cells. A local assessment of the survival rate was calculated for each cell based on the development samples belonging to that cell. Confidence bounds were derived at a $95\%$ confidence level using the area sample size and the following formula^25^:

2 $$\begin{aligned} width = 2z_{\alpha }\sqrt{\frac{P(1-P)}{N}} \end{aligned}$$

with $\alpha = 1{-}confidence_{level}$, $z_{\alpha }$, the value for 2 normal distribution, *P*, the outcome probability and *N*, the sample size.

Cell sample size directly influenced the cell survival rate. The less populated a cell, the wider the probability confidence interval, and the less reliable the analysis of cell membership. Cells were combined to form three equally populated zones with sample sizes sufficient to bound survival rates’ confidence intervals. These zones were designed to have distinct survival rates. Validation data were projected onto the UMAP projection space to check if distribution patterns observed for development data still held. RW patients were then assigned to their corresponding survival rate zone. Validation data zone assignment was assessed with regards to actual survival.

The model was compared to logistic regression and random forest models. Models were trained on two different subsets of features: all of the baseline features and specifically age and baseline ALSFRS features. Models were trained on development data and tested on validation data. The number of True Positives (TP), False Positives (FP), False Negatives (FN) and True Negatives (TN) were reported for each model. The following classification metrics were used: accuracy ($\frac{TP+TN}{TP+TN+FP+FN}.$), precision (or positive predictive value = $\frac{TP}{TP+FP}.$), specificity (or true negative rate, selectivity = $\frac{TN}{TN+FP}.$), recall (or sensitivity, true positive rate = $\frac{TP}{TP+FN}.$), balanced accuracy (average of precision and recall = $\frac{Precision+Recall}{2}$.) and F1-measure (harmonic mean of precision and recall = $2\frac{Precision \times Recall}{Precision+Recall}.$). As the model returned a survival probability and not a survival status, the total number of survivors could only be approximated. It was calculated by adding up the number of survivors for each zone which was based on the total number of patients within that zone and the associated survival rate.

## Results

### Analysis of patient characteristics—input feature distribution

**Figure 1:**
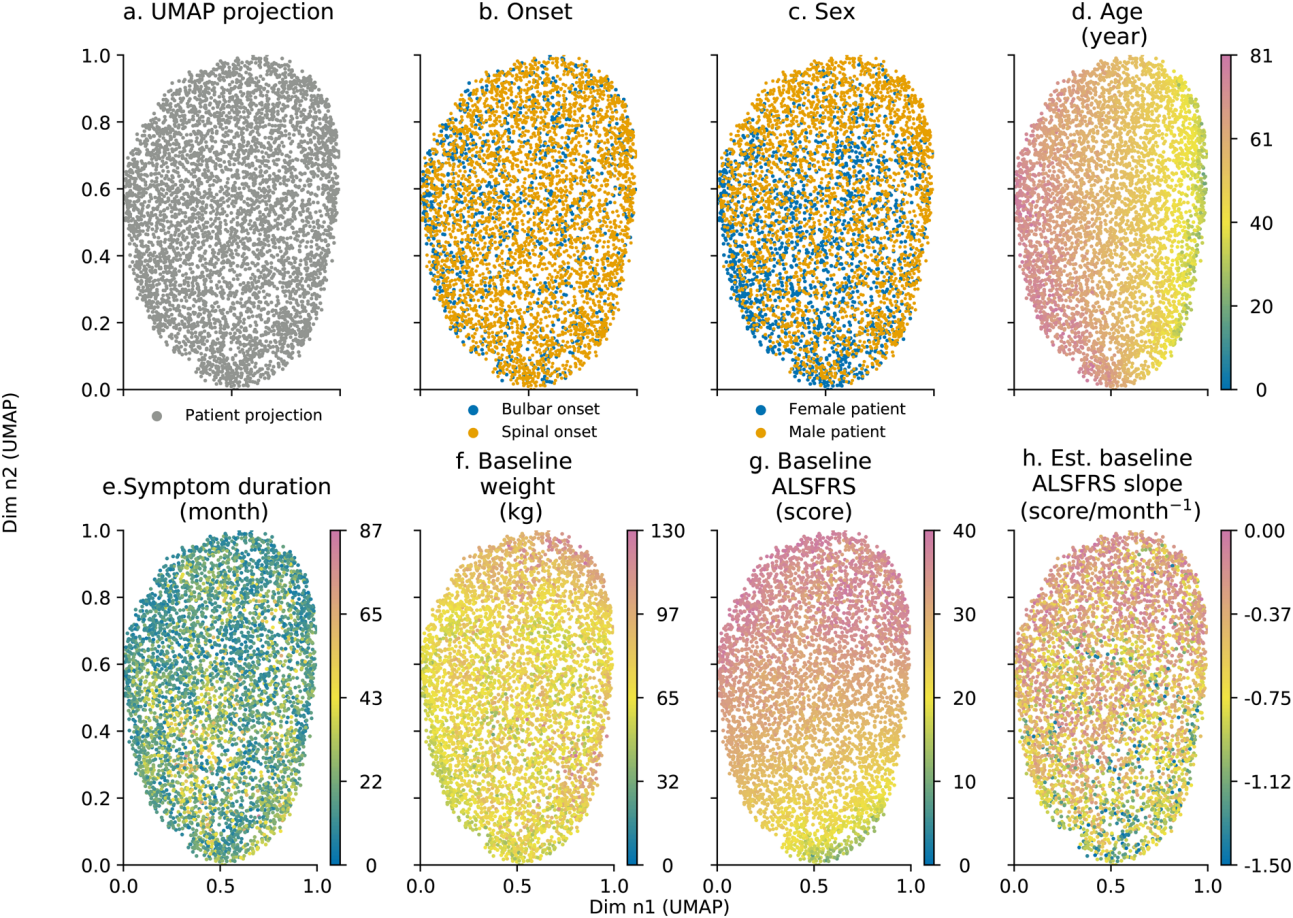
Predictors: onset (**b**), sex (**c**); age (**d**); symptom duration in month (**e**); baseline weight in kg (**f**), baseline ALSFRS score (**g**); and estimated ALSFRS loss rate (**h**) distribution with regards to UMAP projection (**a**). Each point represents an individual patient. Age ranges between 18 and 81 years old (**d**), symptom duration ranges between 0.5 and 87 months (**e**), baseline weight ranges between 30 and 130 kg (**f**), ALSFRS score ranges between 0 and 40 (**g**) and estimated baseline ALSFRS slope ranges between 0.00 and − 1.50 ALSFRS points per month (**h**). Axes are dimensionless and come from UMAP dimension reduction.

Development data were projected using UMAP in a 2D space shown in Fig. 1a. Initial plot of data did not show relevant patient stratification as all patients were clustered together. Plot analysis helps to identify strong correlations between projection and predictors. This was the case for age and baseline ALSFRS scores (Fig. 1d,g respectively) and to a lesser extent for symptom duration and estimated ALSFRS decline rate (Fig. 1e,h respectively). Onset, gender, and baseline weight did not show a high degree of correlation as demonstrated in Fig. 1b,c,f as feature distribution appeared to be random with regards to UMAP projection. Projection data seemed to be independent of cohort membership as patients from each source were evenly distributed in the projection space.

### Analysis of patient outcomes—output feature distribution

**Figure 2:**
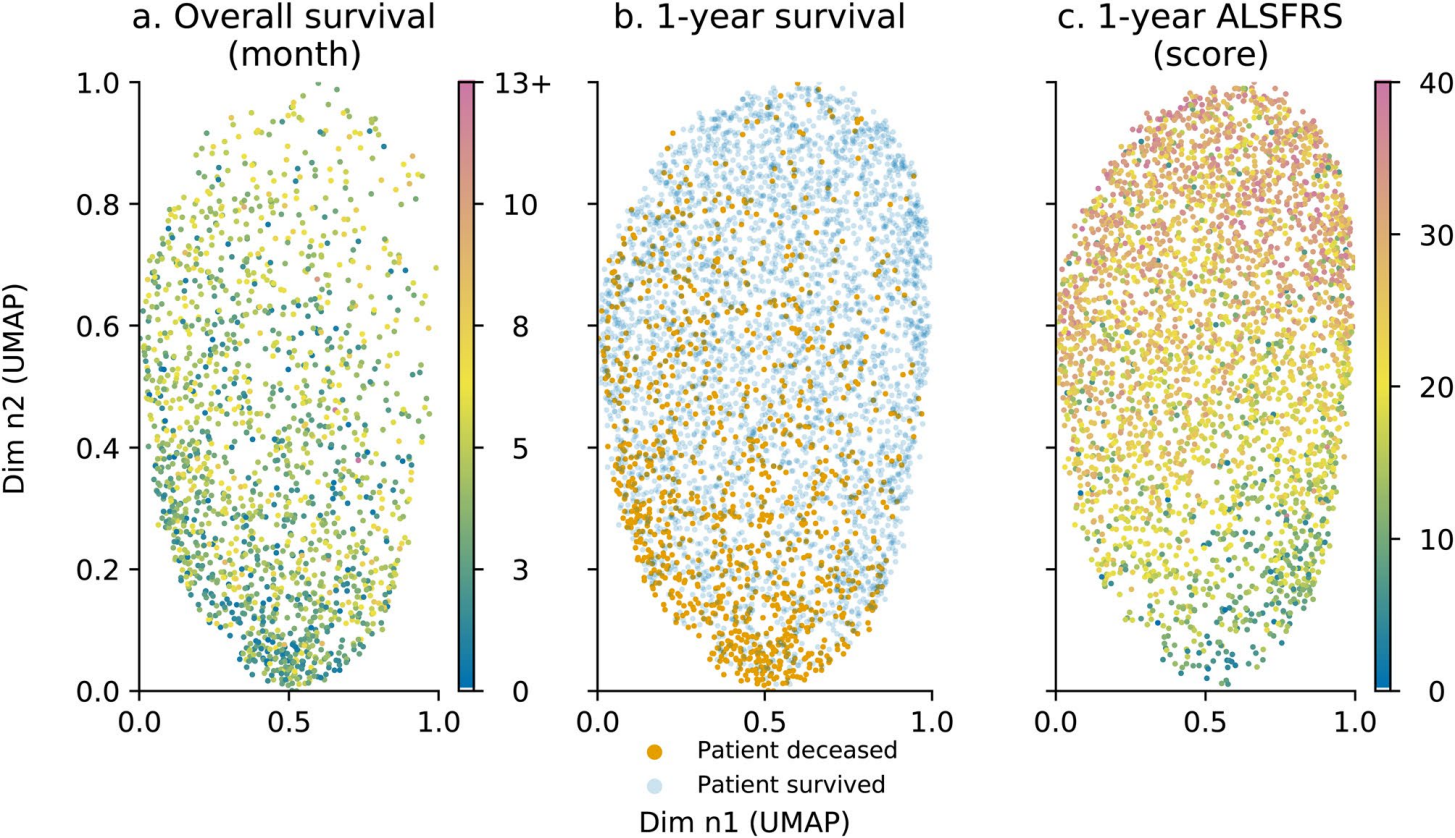
Outcomes: overall survival (**a**); 1-year survival (**b**) and 1-year functional loss (**c**) distribution with regards to UMAP projection in Fig. 1a. Each point represents an individual patient. For overall survival (**a**), survival ranges between 0 and 12 months. 13+ refers to patients whose death date is 13 months or higher. ALSFRS score ranges between 0 and 40 (**c**). For overall survival (**a**) and 1-year functional loss, the data point colour is mapped to a specific time value (for **a**) or ALSFRS score (for **c**). Axes are dimensionless and come from UMAP dimension reduction.

Analysis of UMAP projection with regards to outcome variables showed spatial patterns as presented in Fig. 2. Survival in months is shown in Fig. 2a. Patients with a longer survival (more than 12 months is referred to as the 13+ on the colour map) tended to be located in the upper part of the UMAP projection. 1-year survival led to an uneven patient distribution, as shown in Fig. 2b. Patients deceased within the year tended to concentrate in the lower pane of the UMAP projection which was consistent with the pattern for overall survival. Patients who survived a year tended to spread evenly across the entire projection space. Fig. 2c shows that similarly to 1-year survival, the 1-year ALSFRS score correlated well with the UMAP projection. ALSFRS score patterns differed slightly from 1-year survival as the lower left pane concentrated patients with lowest ALSFRS. Unsurprisingly, the 1-year ALSFRS score, in Fig. 2c, correlated strongly with baseline ALSFRS score, in Fig. 1g.

### Analysis of projection space segmentation—zone division

**Figure 3:**
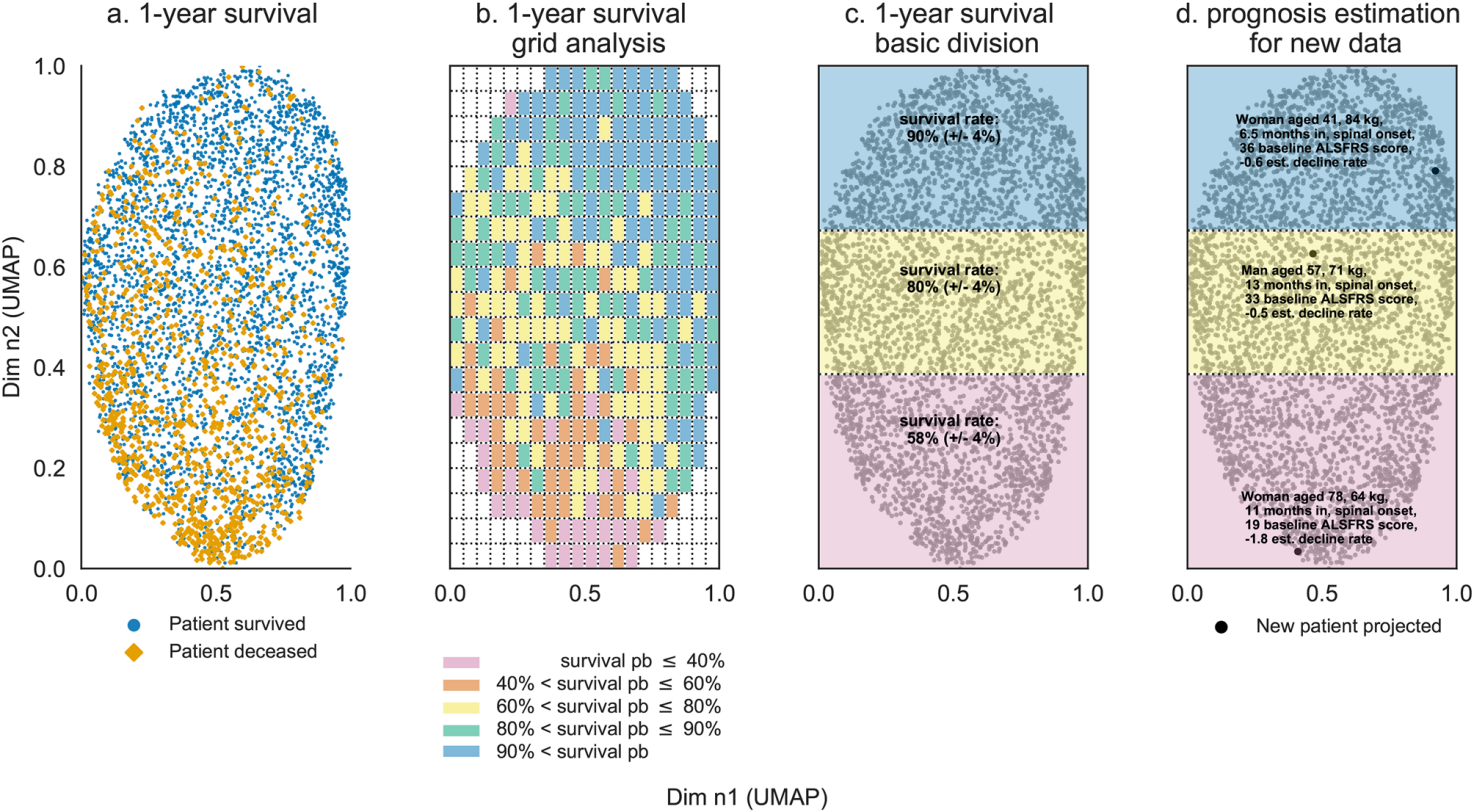
One-year survival projection space segmentation: initial 1-year survival distribution (**a**), projection space division using square cells and survival probability estimation per cell (**b**), resulting projection space division using cell survival probability distribution (**c**), novel patient data projection (**d**). Each point represents an individual patient. The projection space is divided in a square grid (**b**) with each cell having a specific survival rate computed based on patients belonging to that cell (which have either survived or deceased within the year). The overall space is divided in three zones (**c**); the survival rate for each zone is calculated using patients belonging to each zone. Novel patient data is projected into the reduced space and prognosis is estimated based on projection coordinates (**d**). Axes are dimensionless and come from UMAP dimension reduction.

As stated earlier, patients who were not alive at year 1 were mainly located in the lower pane of the projection space as seen in Fig. 3a. Dividing the projection space in square cells helped to unravel local survival patterns as shown in Fig. 3b. Cells in the lower left side of the projection space had survival rates lower than $40\%$. As average sample size within each cell is below 25, confidence intervals were approximately $\pm 30\%$ minimum with survival rate between 10 and $70\%$. To ensure statistical significance, a simple division of the UMAP projection space according to the vertical axis was proposed as shown in Fig. 3c. This led to high, intermediate, and low survival rate zones with respectively $90\%$ ($\pm 4 \%$), $8\%$ ($\pm 4\%$) and $58\%$ ($\pm 4\%$) survival rates. Predictors of patient population within each zone are presented in Table 4. Baseline features for the intermediate survival rate zone were similar to overall baseline features. Baseline features for high and low survival rate zones differed significantly from one another. The former had younger patients and patients with higher weight with shorter symptom duration, with less functional disability and lower functional loss rate; while the latter had older patients with lower baseline weight and longer symptom duration, higher functional loss and functional loss rate.

Novel patient data, provided all baseline features are recorded, can be projected in the reduced UMAP space. The corresponding 2D coordinates determine zone membership to one of the three survival rate zones. Zone membership and the spatial positioning within the projection space provide a broad estimate of patient 1-year survival. Three examples are provided for more details and presented in Fig. 3d:

- Patient A (ID 4922) is a 41-years-old woman with a spinal onset, baseline weight is 84 kg, baseline ALSFRS score is 36, symptom duration is estimated at 6.5 months, hence estimated baseline ALSFRS decline rate is assessed at − 0.6 ALSFRS points per month. This information is used to compute the spatial coordinates of patient A within the UMAP projection space. Patient’s A spatial coordinates in the UMAP projection space are (0.92, 0.79), which fall into the high survival rate zone. Patient A has a resulting 1-year survival rate estimate of $90\%$.
- Patient B (ID 429) is a 57-years-old man with a spinal onset, baseline weight is 71 kg, baseline ALSFRS is 33, symptom duration is estimated at 13 months, hence baseline estimated ALSFRS decline rate is assessed at around − 0.5 ALSFRS points per month. This information is used to compute the spatial coordinates of patient B within the UMAP projection space. Patient’s B spatial coordinates in the UMAP projection space are (0.46, 0.62) which fall into the intermediate survival rate zone. Patient B has a resulting 1-year survival rate estimate of $80\%$.
- Patient C (ID 2816) is a 78-years-old woman with a spinal onset, baseline weight is 64 kg, baseline ALSFRS is 19, symptom duration is estimated at 11 months, hence baseline estimated ALSFRS decline rate is assessed at around − 1.8 ALSFRS points per month. This information is used to compute the spatial coordinates of patient C within the UMAP projection space. Patient’s C spatial coordinates in the UMAP projection space are (0.41, 0.03) which fall into the intermediate survival rate zone. Patient C has a resulting 1-year survival rate estimate of $58\%$.

Subsequent analysis of patients’ A, B and C status after one year are that patient A and B survived a year while patient C died within the first year. A refined division of the projection space was also carried out and is presented in the supplementary information section.

**Table 4:** Predictor distribution per survival area.

Feature	PRO-ACT	Exonhit	Trophos	Real world	Overall
Initial sample size (n)	10,723	400	512	1,377	13,012
Gender	$0 \%$	$0 \%$	$0 \%$	$0 \%$	$0 \%$
Onset	$12 \%$	$0 \%$	$0 \%$	$2 \%$	$10 \%$
Age	$28 \%$	$0 \%$	$0 \%$	$0 \%$	$23 \%$
Symptom duration	$36 \%$	$0 \%$	$0 \%$	$0 \%$	$30 \%$
Baseline weight	$39 \%$	$3 \%$	$1 \%$	$3 \%$	$33 \%$
Baseline height	$38 \%$	$0 \%$	$100 \%$	$3 \%$	$35 \%$
Baseline ALSFRS	$36 \%$	$2 \%$	$0 \%$	$0 \%$	$30 \%$
Baseline ALSFRS upper limb sub-score	$39 \%$	$0 \%$	$0 \%$	$0 \%$	$36 \%$
Baseline ALSFRS lower limb sub-score	$39 \%$	$0 \%$	$100 \%$	$0 \%$	$36 \%$
Baseline ALSFRS bulbar sub-score	$39 \%$	$0 \%$	$100 \%$	$0 \%$	$36\%$
Baseline ALSFRS respiratory sub-score	$39 \%$	$0 \%$	$100 \%$	$0 \%$	$36 \%$
Baseline ALSFRS trunk sub-score	$39 \%$	$0 \%$	$100 \%$	$0 \%$	$36 \%$
Baseline pulse	$32 \%$	$1 \%$	$100 \%$	$100 \%$	$41 \%$
Baseline diastolic blood pressure	$32 \%$	$1 \%$	$0 \%$	$100 \%$	$37 \%$
Baseline systolic blood pressure	$32 \%$	$1 \%$	$0 \%$	$100 \%$	$37 \%$
Baseline vital capacity (L)	$23 \%$	$1 \%$	$0 \%$	$100 \%$	$29 \%$
Baseline vital capacity ($\%$)	$10 \%$	$1 \%$	$0 \%$	$100 \%$	$19\%$
Survival (month)	$68 \%$	$55 \%$	$80 \%$	$66 \%$	$68 \%$
1-year survival	$46 \%$	$15 \%$	$16 \%$	$59 \%$	$45 \%$
1-year ALSFRS	$66 \%$	$42 \%$	$30 \%$	$75 \%$	$65 \%$
Overall missing ratio	$35 \%$	$6 \%$	$41 \%$	$35 \%$	$34 \%$
Overall predictor missing ratio	$30 \%$	$1 \%$	$41 \%$	$30 \%$	$30\%$
Overall outcome missing ratio	$60 \%$	$37 \%$	$42 \%$	$67 \%$	$59\%$
Final sample size for 1-year survival (n)	3,971	172	646	431	5,220

### Analysis of the model with additional data—external data testing

**Figure 4:**
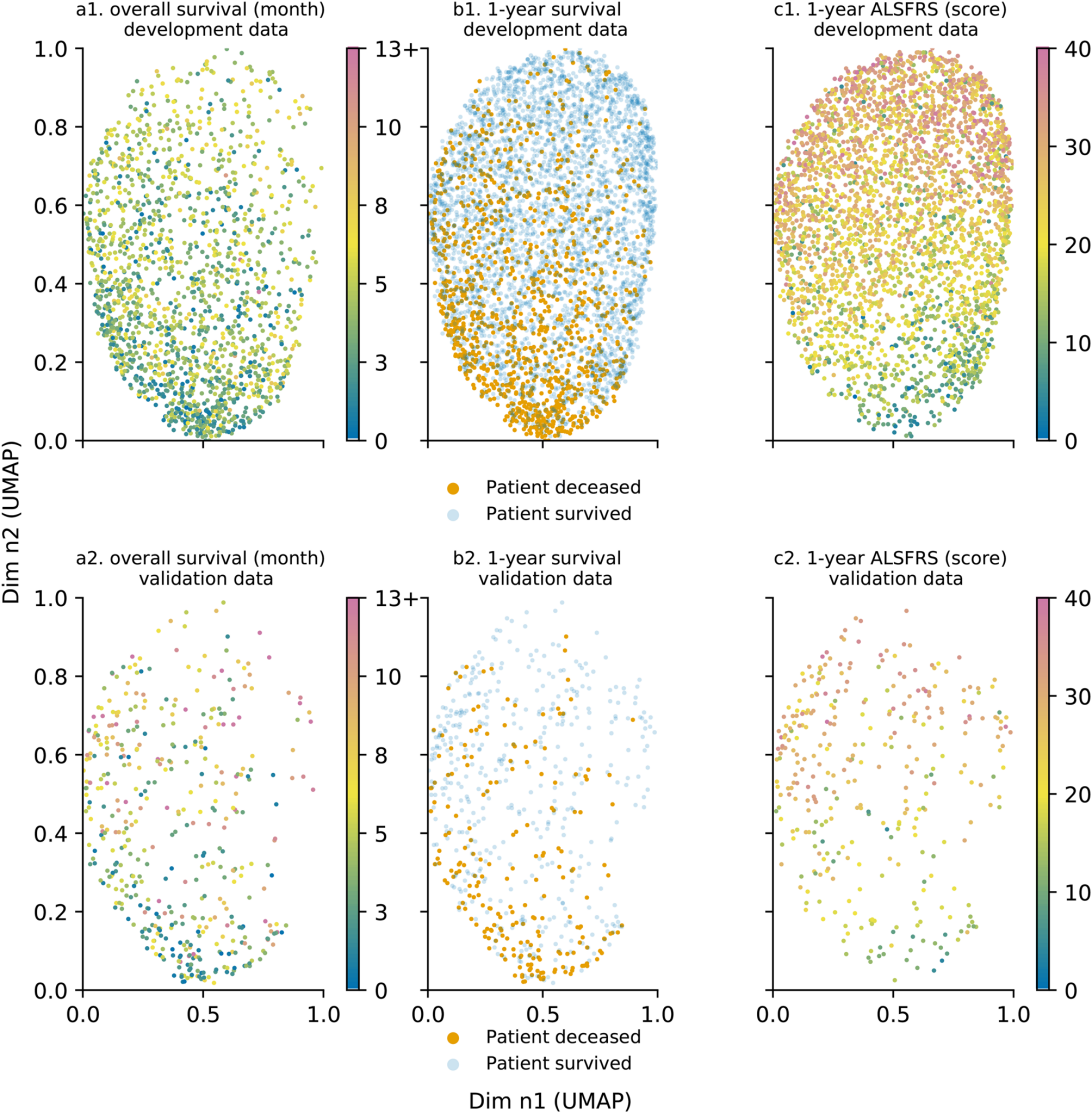
Outcomes with regards to UMAP projection for development and validation data: overall survival for development (**a.1**) and validation (**a.2**) data, 1-year survival for development (**b.1**) and validation data (**b.2**) and 1-year functional loss for development (**c.1**) and validation data (**c.2**) (for overall survival, 13+ refers to patients whose death date is 13 months or more). Each point represents an individual patient. For overall survival (**a**), survival ranges between 0 and 12 months. 13+ refers to patients whose death date is 13 months or higher. ALSFRS score ranges between 0 and 40 (**c**). For overall survival (**a**) and 1-year functional loss, the data point colour is mapped to a specific time value (for **a**) or ALSFRS score (for **c**). Axes are dimensionless and come from UMAP dimension reduction. (**a.1**), (**b.1**) and (**c.1**) represent development data plots; (**a.2**), (**b.2**) and (**c.2**) represent validation data plots.

The prognosis model was assessed using external data. Patient distribution within the projection space was examined with regards to outcome variables. The different trends for outcome variables identified in Fig. 2 remained valid with patient distribution being uneven for patients who die within one year. Patients with a shorter survival tended to concentrate in the lower pane of the projection, as shown in Fig. 4a, as did patients who do not reach the 1-year milestone in Fig. 4b. Patients were also distributed similarly based with regards to the functional loss pattern identified earlier. Patients were distributed according to their impairment after one year of follow up. Patients suffering from a stronger functional loss were located in the lower-left part of the projection, as presented in Fig. 4c. Additional information on differences between development and validation data using the Kullback-Leibler divergence and complementary figures on distribution comparisons are presented in the supplementary information section.

### Zone division—external data evaluation

Patient distribution within the three zones is presented in Table 5. $42\%$ of the RW patients went within the low survival rate zone, while $25\%$ go within the high survival rate zone, and the $33\%$ remaining to the non-informative intermediate survival rate zone. The overall survival rate of the RW patient dataset was $67\%$. Measured survival rates within the low, intermediate, and high survival rate zones were respectively $48\%$, $76\%$, and $88\%$. Patients in the low survival rate group had a poorer survival rate than observed with trial data. Adding 646 patients reduced the overall confidence bound for survival relatively by $6\%$ (from $2.43\%$ to $2.28\%$).

**Table 5:** Real-world validation data distribution per survival area.

Group	Deceased	Survived	Count per zone	Percent per zone
High survival rate zone	20	140	160	$25\%$
Intermediate survival rate zone	51	160	211	$33\%$
Low survival rate zone	142	133	275	$42\%$
Count per status	213	433	646	
Percent per status	$33\%$	$67\%$		

The model was compared to logistic regression and random forest models. Results are presented in Table 6. $90\%$ of the 160 patients associated with the high survival rate zone were labelled as survivors (144). $80\%$ of the 211 patients belonging to the intermediate survival rate zone were labelled as survivors (159). $58\%$ of the 275 patients assigned to the low survival rate zone were labelled as survivors (160). Overall 473 patients were predicted to survive, 173 were predicted to die. 433 patients actually survived and 213 died. Performance assessment is approximated based these figures. Hence 433 survivors (TP) and 173 deceased patients (TN) were predicted correctly while 40 patients were wrongly labelled as survivors (FP). Our model obtained classification metrics higher than the other models’, specifically with regards to the F1-measure and balanced accuracy metric where our model reached respectively 96$\%$ and 91$\%$ scores in opposition to the other models averaging around $50\%$ and $65\%$ scores.

**Table 6:** Model comparison on validation data.

Model	TP	FP	FN	TN	Accuracy (%)	Precision (%)	Specificity (%)	Recall (%)	Balanced accuracy (%)	F1 measure (%)
LR 2 features	89	124	58	375	72	42	75	61	68	49
LR 7 features	100	113	64	369	73	47	77	61	69	53
RF 2 features	85	128	96	337	65	40	72	47	60	43
RF 7 features	119	94	104	329	69	56	78	53	66	55
Proposed Model	433	40	0	173	94	91	81	1000	91	96

LR, RF and Proposed Model respectively stand for Logistic Regression, Random Forest and UMAP combined to spatial division.

## Discussion

Our study demonstrated the utility of UMAP for survival analysis in ALS. We have successfully applied this non-linear dimension reduction method to ALS clinical trial data to predict overall survival, 1-year survival and 1-year functional loss. Our results showed that limited patient information, collected early in the course of the disease, was sufficient to obtain a relevant low-dimensional patient projection with regards to key outcome variables (survival and functional loss). These input features correlated with the different outcomes of interest, thus explaining the observed distribution patterns. These correlations persisted for external RW patients. One-year survival patient distribution patterns were used to identify zones with distinct survival rates. We proposed a simple 1-year survival estimation model which fared well against the tested machine learning models although performance metrics could only be grossly approximated. The benefit of our approach with regards to standard machine learning methods is threefold. First, our model is simple; it uses only simple probabilities and readily available clinical features. Second, we limit prognosis error by providing a coarse prognosis estimate. Third, our model is easily updated and improves with additional data. No learning was required for our model to work as UMAP is a dimension reduction method. Given dimension reduction was performed on baseline features, projection analysis can be extended to other prognosis outcomes, namely functional loss or clinical staging, and different time frames.

As this study evaluated pre-existing datasets we faced a number of constraints. PRO-ACT data are not uniformly recorded; for instance, vital capacity may be available in litres or percent, and slow and forced vital capacities are inconsistently documented. Units for weight are not clearly labelled as pounds or kilograms. A weight value of 99 without an associated unit may equally be interpreted as kilograms or pounds. These inconsistencies concern $26\%$ of PRO-ACT patients. Inclusion criteria for all datasets pooled within PRO-ACT are not comprehensively documented; 6 out the 23 pooled clinical trial names were not disclosed. Available trial data also suffer from inclusion bias, as patients with marked cognitive or behavioural impairment often face worse prognosis^26–28^, and are often excluded from or drop out of clinical trials.

Missing data imputation was omitted and our model was trained solely on complete case samples. Although generally recommended in medical settings, data imputation seemed hazardous in this specific data context, specifically working with PRO-ACT. Multiple imputation methods often assume that the missingness patterns are missing at random, i.e. that they depend on other observed variables in the dataset. This information is difficult to verify and these data imputation methods are often performed on the biggest feature subset available so as to improve the odds of such a hypothesis being true. Given the differences in the data collection process and the limited feature subset shared between the different datasets, data imputation could not have been carried out on the global data structure. Data imputation at a dataset level would not have been productive and would have led to significant additional noise in data given small sample size and significant missing feature ratio for each dataset. Even advanced multiple imputation methods such as Quartagno et al.^29^ which deal with missing data imputation at a study level (for meta-analysis purposes) require knowing the collection process for each study in scope, which we cannot access for PRO-ACT as features could be missing due to loss to follow up or due to clinical trial setup. Furthermore, as UMAP is a neighbourhood-based approach, data imputation can be seen as adding data where it is missing. This would have induced sample similarity in cases where little information was known on the subjects, creating visual artefacts of similar patients within the projection space and adding significant bias to the visual representation. Our spatial distribution approach would have had a more limited performance had we worked with imputed data that would have artificially created spatial proximity.

Another data constraint was that lack of availability of established prognostic indicators in at least one of the four datasets, such as ALSFRS sub scores, cognitive profile, Riluzole intake, vital capacity^30^, time to generalisation^31^ or weight loss, which is considered more relevant than absolute weight at baseline^32^. This limited the model’s ability to discriminate patients within the projection space. Additional clinical features, such as upper or lower limb onset, upper or lower motor neuron predominance, may be potential predictors to improve our model further. The inclusion of biological, genetic^33^, and imaging features^14,34^ are likely to have improved current prognosis modelling^35^. In our study, overall survival was only regarded as a secondary outcome as global survival was not available in most cases. Analysis of overall survival would not have led to accurate results given the available data is predominantly censored after trial end. As overall survival prediction remains key and 1-year survival, a substitute target, it seemed relevant to analyse how overall survival correlated with UMAP projection coordinates. Given our data, 1-year survival was a good proxy of overall survival.

Feature processing excluded dealing time-resolved features in a time-series manner, comparable to past ALS prognosis studies^15,36–38^. As such, feature processing and model design was simplified. Time-series information, specifically with regards to ALSFRS, was obtained using intercept and slope values. As such, we did not intend to carry out a statistical analysis of data using traditional Kaplan Meier (for 1-year survival) or Cox regression (for functional loss) approaches that factor in time and censoring. A Kaplan–Meier approach can provide an interesting overview of the outcome with regard to time but never at a patient level which is the approach we wished to explore.

As a non-linear unsupervised learning model, UMAP can capture and characterise complex relationships between predictors. UMAP is more than a data visualisation method: the projection space preserves distances, density and neighbourhoods which allow manipulation of projected data through spatial analysis or clustering methods. However, it is a black-box approach. Model interpretability cannot be obtained: the explicit relationship between UMAP input and output variables remains unavailable. Analysis of input feature distribution in the UMAP projection gives a broad overview of variable importance with regards to the projection. Data is projected in a reduced space with interesting data distribution and preserved input space proprieties. UMAP provides the foundation to develop our prognosis model which derived from UMAP space segmentation. Our model combined UMAP with a simple spatial division in order to leverage observed correlations between projection features and the primary outcome. As such, similarly to other machine learning models, UMAP identifies underlying data correlations but cannot reveal causal relationships. Nevertheless, our model provides confidence intervals which most machine learning techniques such as random forest, boosting or neural network methods do not ordinarily provide. This additional information can help clinicians to evaluate prognosis in finer detail.

ALS prognosis modelling has been already been extensively researched in the past. Random forest models were frequently tested^15,36,37,39–41^, repeatedly outperforming other machine learning models. As logistic regression is a probabilistic model, it seemed interesting to compare our model with these two machine learning models. Given the strong correlation between age and baseline ALSFRS features and projection space coordinates, evaluating model performance on this feature subset was also valuable. Given the imbalance with regards to the outcome (as $75\%$ of patients survived 12 months), accuracy alone would not have been a reliable performance metric. Precision and recall metrics provided a finer understanding of model weaknesses and strengths. As performance metrics were calculated differently for our model and the other machine learning models, where individual predictions were available for all patients, performance results should be viewed with caution.

Given the cell sample size, the estimated survival probability for each cell was not directly used for prognosis estimation, as the confidence interval was not narrow enough. Although each cell carried limited survival information on its own; combined, they were useful in understanding the differences in spatial distribution. Sample size was crucial as it directly influenced the level of detail for the projection space division. A larger data sample would be required to define more zones with distinct survival rates. Dividing the projection space in three was deemed the most appropriate approach given the patient distribution and sample size. Based on the available data, we had to deal with the trade-off between prognosis personalisation and narrow confidence bounds for survival. Testing on external RW data was necessary to assess model ability to scale up and model validity as it was designed using trial patients. Only minor differences were observed when assessing zone membership. A large number of patients were assigned to the low survival rate zone. This is clearly explained by the fact that clinical trials have inclusion criteria which select less severe patients. Additional RW data could correct this bias and limit the resulting over-optimistic prognosis it entails.

In conclusion, we have successfully implemented a simple 1-year survival model partially based on a novel non-linear unsupervised learning method. Further work will be needed to extend our analyses to other prognosis outcomes, such as functional loss and clinical staging systems. Given the relatively low incidence of ALS compared to other neurodegenerative conditions, robust international collaborations are necessary to collect large datasets and build precision models^42^. Notwithstanding the constraints of the available data, we have demonstrated that combining UMAP with a probabilistic and spatial distribution analysis, important correlations can be unravelled.

## Supplementary information

Supplementary Information.

## Data Availability

Anonymised data are freely accessible from the public database of the Northeast ALS Consortium. Statistical code are shared on the following github: $alsparis/als\_survival\_prognosis$.
