# Supplementary material for: Development and validation of a 1-year survival prognosis estimation model for Amyotrophic Lateral Sclerosis using manifold learning algorithm UMAP
Source: Sci Rep. 2020 Aug 7;10:13378. doi: 10.1038/s41598-020-70125-8 (PMC7414917; doi:10.1038/s41598-020-70125-8)
Supplement: Supplementary file 1 — Supplementary Information. [file 41598_2020_70125_MOESM1_ESM.pdf]

Development and validation of a 1-year survival prognosis estimation model for Amyotrophic Lateral Sclerosis using manifold learning algorithm UMAP

Vincent Grollemund[1,2,*], Gaétan Le Chat[2], Marie-Sonia Secchi-Buhour[2], François Delbot[1,3], Jean-François Pradat-Peyre[1,3], Peter Bede[4,5,6], and Pierre-François Pradat[4,5,7]

[1]Sorbonne Université, Laboratoire d'Informatique de Paris 6, Paris, 75005, France
[2]FRS Consulting, Paris, 75009, France
[3]Nanterre Université, Modal'X, Nanterre, 92014, France
[4]Sorbonne Université, Laboratoire d'Imagerie Biomédicale, Paris, 75005, France
[5]Pitié-Salpêtrière University Hospital, APHP, Département de Neurologie, Paris, 75013, France
[6]Trinity College, Computational Neuroimaging Group, Dublin, D02 PN40, Ireland
[7]Antnagelvin Hospital, Northern Ireland Center for Stratified Medecine, Biomedical Sciences Research Institute Ulster University, C-TRIC, Londonderry, BT47 6SB, United Kingdom
[*]vincent.grollemund@lip6.fr

ABSTRACT

Amyotrophic Lateral Sclerosis (ALS) is an inexorably progressive neurodegenerative condition with no effective disease modifying therapies. The development and validation of reliable prognostic models is a recognised research priority. We present a prognostic model for survival in ALS where result uncertainty is taken into account. Patient data were reduced and projected onto a 2D space using Uniform Manifold Approximation and Projection (UMAP), a novel non-linear dimension reduction technique. Information from 5,220 patients was included as development data originating from past clinical trials, and real-world population data as validation data. Predictors included age, gender, region of onset, symptom duration, weight at baseline, functional impairment, and estimated rate of functional loss. UMAP projection of patients shows an informative 2D data distribution. As limited data availability precluded complex model designs, the projection was divided into three zones with relevant survival rates. These rates were defined using confidence bounds: high, intermediate, and low 1-year survival rates at respectively 90% (±4%), 80% (±4%) and 58% (±4%). Predicted 1-year survival was estimated using zone membership. This approach requires a limited set of features, is easily updated, improves with additional patient data, and accounts for results uncertainty.

Supplementary information

Additional information on datasets

The first dataset, referred to as 'Trophos', was a 2014 double-blind, randomised, placebo-controlled phase II-III clinical trial for olesoxime, a drug produced by Trophos[1]. The trial lasted 18 months; recruitment started on May 4th 2009 and ended on March 31st 2010. The final follow up was on September 15th 2011. Data were collected on 512 patients with clinical and biological data. Patients were recruited from 15 tertiary referral centres in 5 European countries. Inclusion criteria included 'probable' or 'definite' ALS on the El Escorial criteria, a slow vital capacity above or equal to 70% of the height-adjusted normative value, age between 18 and 80 years old, symptom duration between 6 and 36 months and Riluzole treatment (50 mg of Riluzole, twice a day for at least a month). The trial was conducted according to the European guidelines for Good Clinical Practice.

The second dataset, named 'Exonhit', was a 2006 double-blind randomised, place-controlled phase II-III clinical trial for pentoxifylline, a drug developed by Exonhit Pharma[2]. The trial lasted 18 months, with recruitment starting on October 3rd 2002 and ending on February 12th 2003. Follow ended on August 6th, 2004. Data were collected on the 400 patients and limited to clinical and muscle strength testing data. Patients were recruited from 12 tertiary referral care centres from 4 countries. Patients were selected based on a 'probable' or 'definite' ALS using the El Escorial criteria, age between 18 and 80, a symptom duration between 6 and 47 months and Riluzole treatment (established on 50 mg Riluzole, twice a day). The trial was conducted according to European guidelines for Good Clinical Practice.

The third database is PRO-ACT, which is funded by the ALS Therapy Alliance and released in 2012 as part of the DREAM Phil Bowen ALS prediction Prize4Life competition. It consists of pooled data from 16 completed phase II-III clinical trials and

one observational study[3]. Included clinical trials were conducted between 1990 and 2010 and lasted on average 12 months. Core patient data included clinical and biological lab results data. In December 2015, data from 5 clinical trials were added to PRO-ACT without additional information, totalling 22 different clinical trial sources and 10,723 patients. Patient age ranged from 18 to 88, 82% were on Riluzole therapy (with variable dosage), but no El Escorial categorisation or individual vital capacity values were made available.

The fourth dataset is population-based and contains RW patient data. Data were obtained from the database of the Paris tertiary referral centre for ALS (Pitié Salpêtrière Hospital – Assistance Publique des Hôpitaux de Paris, France) between September 1999 and April 2008. The initial patient sample size was 1,377 and included data on baseline ALSFRS sub-scores, age, time interval since symptom onset, El Escorial criteria and muscle strength values.

As patient treatment is not relevant for our current work; please refer to corresponding clinical trial articles for additional information.

Additional information on UMAP

Supervised learning models usually require large amounts of data to avoid overfitting and lack of generalisation, which aren't available in ALS research. Unsupervised learning methods have the advantage of capturing distribution patterns without data implications. Standard linear methods such as Principal Component Analysis (PCA)[4] have been used in ALS for gene expression analysis[5]. Unfortunately, these conventional linear-based methods are not capable of describing non-linear relationships and have underperformed in this study context. Non-linear methods provide new modelling possibilities given their comprehensive ability to describe data correlations and have successfully been tried out for ALS phenotype identification on clinical trial data[6] with the current state-of-the-art manifold learning model, t-Student Stochastic Neighbour Embedding (t-SNE)[7]. UMAP outperforms t-SNE on the following aspects: it scales with regards to complexity (calculation-wise), allows dimension reduction for other purposes than visualisation (i.e. dimensions can be larger than 3), has a convex cost function (where t-SNE has a non-convex cost function that leads to initialisation based results) and preserves neighbourhood, distances and density (and not only neighbourhood like t-SNE)[8]. All these assets make UMAP more suitable for clustering at a later stage than t-SNE.

UMAP is neighbourhood-based and works in two steps. First, a compressed embedding of the input space is built through topological analysis of the data structure using simplexes[1]. The compressed embedding is a simplicial simplex which can be seen as a neighbourhood graph of the input data that is built from the open cover[2] of the simplexes. Second, a low-dimensional (in our case two-dimensional) data embedding is found through a cross-entropy[3] optimisation process. UMAP preserves data neighbourhoods, distances and density. The initial modelling step depends on whether the algorithm should focus on preserving the local or global input data structure. Data structure is estimated according to the size of the neighbourhood investigated. The second compression step is mainly defined using two parameters which are the output dimension size and the minimum distance permitted between two points in the output space, i.e. how compact the output projection can be.

Direct model specification is not possible as the UMAP projection function is a black-box approach. That being said, the UMAP projection function can be stored and used afterwards on new data. As it is a black-box approach, projection features cannot be analysed to provide any interpretability. Output dimension analysis, as commonly performed for PCA, cannot be carried out. Analysis of input feature distribution in the UMAP projection space is an alternative as it gives a broad overview of variable importance with regards to the projection. In **Figure 1**, age and baseline ALSFRS score appear to be the variables which matter most distance-wise in the output space. Both variables have a global incidence on the overall UMAP projection distribution. Other variables, which show a weaker or limited impact on the overall UMAP projection distribution, may have a more local impact in the output space distance-wise.

Performance is directly related to the observed sample size (and its ability to represent the overall ALS patient population) as, the more data collected, the finer the split in the input space with a controlled confidence bound. UMAP was performed on a 2D plane as the 1D projection led to uninterpretable results and the 3D projection led to results similar to those observed in a 2D setting (with a potato-shaped form). Given the additional dimension, data density was lower and projection analysis and partitioning was more complex so the 2D projection was preferred. Given the current collected dataset size, we could not explore finer splits without a significant increase of the confidence bound. As more data gets collected, a finer division of the projection space can be expected and a precision medicine approach can be implemented to provide clinicians with more distinct patient divisions. Model updating is straightforward as novel data points can be projected onto the 2D space using the learnt projection function.

[1]In geometry, a simplex is defined as a set of points, where none is a barycentre of the remaining points. The convex hull of these points corresponds to the face of the simplex. In simpler terms, a n-simplex can be thought of as the generalisation of a triangle in the nth dimension.

[2]An open cover is essentially just a family of sets whose union is the whole space[9].

[3]In machine learning, cross entropy is frequently used as a cost function to compare two probability distributions (p,q): p is optimised to approximate q the fixed target distribution.

UMAP projection is fitted using the clinical trial data. UMAP projection for additional data is not computed using the UMAP package as proper mixed data type management (when dealing with both categorical and numerical features) has yet to be implemented in the official UMAP package. As a proxy, UMAP projection is learnt using a random forest model (with 10 trees). The random forest model serves as a proxy for now to estimate the 2D mapping of additional samples. Our random forest model reached a 99.3% coefficient of determination (R2) score. The random forest was trained using 80% of the clinical trial data and was tested using the remaining 20%.

Additional information on distribution differences

Differences in distribution between the development and validation datasets have been analysed with regards to each outcome in scope, for different outcome values. These were survival or death for 1-year survival, ALSFRS range for functional loss and survival time range for overall survival. Kullback-Leibler (KL) divergence for development and validation distributions were calculated for the different outcomes and different zone memberships as detailed in **Table 7**. KL divergence between the validation and development distributions for patients within the low survival rate zone with a 1-year ALSFRS higher than 30 was not calculated due to sample size for the development distribution. Visual differences for these subsets are presented in **Figure 5** and **Figure 6**. Distribution patterns with regards to 1-year survival for patients that died appeared similar, as presented in **Figure 5b**. For patients that survived, in **Figure 5c**, patients seemed to concentrate on one side of the projection pane. Distribution differences, given the discrepancies in sampling size and biases, are difficult to explain. Distribution patterns with regards to overall survival loss seemed similar with regards to the 4 different survival intervals as shown in **Figure 5d** to **5g**. Distribution differences appeared stronger for patients with weaker functional loss, as shown in **Figure 5h** and **5k** as our validation data had weaker patients. The left shift observed for 1-year survival was also observed. A more refined analysis of distribution patterns for 1-year survival is presented in **Figure 6** with the spatial division proposed for our model. Overall, spatial differences corresponded to the left shift previously identified. The limited sampling size for deceased patients within the high survival rate zone can partially explain how the differences in distributions according to the x-axis seemed so strong.

Additional information on statistical testing

Significance testing was performed (using an F-test/ANOVA) and identified significant differences in means for all features except gender and onset, as shown in **Table 8**. Significance testing was also tested (using an F-test/ANOVA) on clinical trial data only and identified significant differences in means for all features but gender, onset and age, as presented in **Table 9**. ANOVA testing assumptions regarding homogeneity of variance and normality were, however, not met, as shown in **Table 10**. Patient samples are however independent. Differences in distributions for all four datasets are not relevant with regards to model design. They do not impact non-linear dimension reduction methods, and in our case UMAP, as they do not require specific distribution assumptions for data analysis. Showing that all datasets are equivalent has a limited added value with regards to model generalisability as collected data is biased[10]. Differences in means, for clinical trial data, for all features except gender, onset, and age can be partially explained by discrepancies in trial inclusion criteria. Dissimilarities between clinical trial and RW data were expected due to patient selection bias in clinical trials. Inclusion of Trophos and Exonhit trials are meant to broaden patient scope (i.e. case typologies). As in clinical trials, patient selection is carried out to maximise patient survival till clinical trial end. This overestimates patient survival. Adding additional sources of data improves overall model relevance.

Statistical testing is carried out using the analysis of variance test (ANOVA), a generalisation of t-test testing to more than 2 distributions. The null hypothesis H0 is that each tested distribution has the same distribution mean. Rejecting the null hypothesis implies that there exists a significant difference between one or more distribution means.

Cohen's rule of thumb for effect size (η^2) analysis: 0.01 for small, 0.06 for medium and 0.14 and more for large.

Three assumptions need to be tested before carrying out ANOVA testing:

- Normality (tested on residuals of the ordinary least square model which fits the outcome (predictor) depending on the source using the Shapiro test);
- Homogeneity of variance (no differences in distribution variances) using Levene's test;
- Independent observations.

Table 7. Kullback-Leibler divergence for validation and development datasets.

Outcome	Outcome value	Zone	n	KL_x	KL_y
Overall			4,574	4.2E-01	3.7E-01
1-year survival	Deceased		1,085	3.6E-01	5.7E-01
1-year survival	Survived		3,489	4.2E-01	2.5E-01
1-year survival	Deceased	Low survival rate zone	633	2.5E-02	4.1E-02
1-year survival	Survived	Low survival rate zone	892	2.9E-02	2.2E-02
1-year survival	Deceased	Intermediate survival rate zone	305	6.3E-02	2.4E-03
1-year survival	Survived	Intermediate survival rate zone	1,219	6.5E-02	2.2E-03
1-year survival	Deceased	High survival rate zone	147	2.9E-02	1.1E-03
1-year survival	Survived	High survival rate zone	1,378	3.2E-02	1.0E-03
Overall survival	Survival $\leq$ 3 months		129	3.2E-01	6.3E-01
Overall survival	3 < Survival $\leq$ 6 months		237	3.1E-01	6.6E-01
Overall survival	6 < Survival $\leq$ 12 months		710	4.3E-01	4.8E-01
Overall survival	Survival > 12		536	4.4E-01	3.7E-01
Overall survival	Survival $\leq$ 3 months	Low survival rate zone	86	2.3E-02	5.5E-02
Overall survival	3 < Survival $\leq$ 6 months	Low survival rate zone	149	2.4E-02	3.9E-02
Overall survival	6 < Survival $\leq$ 12 months	Low survival rate zone	392	3.1E-02	3.4E-02
Overall survival	Survival > 12 months	Low survival rate zone	231	2.7E-02	2.9E-02
Overall survival	Survival $\leq$ 3 months	Intermediate survival rate zone	31	6.6E-02	2.6E-02
Overall survival	3 < Survival $\leq$ 6 months	Intermediate survival rate zone	71	5.2E-02	2.5E-03
Overall survival	6 < Survival $\leq$ 12 months	Intermediate survival rate zone	201	7.4E-02	2.4E-03
Functional loss	ALSFRS $\leq$ 10			3.6E-01	5.6E-01
Functional loss	10 < ALSFRS $\leq$ 20			3.3E-01	3.4E-01
Functional loss	20 < ALSFRS $\leq$ 30			5.4E-01	1.1E-01
Functional loss	30 < ALSFRS			4.7E-01	3.2E-02
Functional loss	ALSFRS $\leq$ 10	Low survival rate zone		2.8E-02	4.0E-02
Functional loss	10 < ALSFRS $\leq$ 20	Low survival rate zone		2.3E-02	1.9E-02
Functional loss	20 < ALSFRS $\leq$ 30	Low survival rate zone		3.6E-02	6.9E-03
Functional loss	30 < ALSFRS	Low survival rate zone	7	NA	NA
Functional loss	ALSFRS $\leq$ 10	Intermediate survival rate zone	358	5.7E-02	2.5E-03
Functional loss	10 < ALSFRS $\leq$ 20	Intermediate survival rate zone	334	4.6E-02	2.7E-03
Functional loss	20 < ALSFRS $\leq$ 30	Intermediate survival rate zone	615	6.7E-02	2.3E-03
Functional loss	30 < ALSFRS	Intermediate survival rate zone	165	1.0E-01	1.3E-03
Functional loss	ALSFRS $\leq$ 10	High survival rate zone	168	4.1E-02	1.0E-03
Functional loss	10 < ALSFRS $\leq$ 20	High survival rate zone	178	2.7E-02	7.3E-04
Functional loss	20 < ALSFRS $\leq$ 30	High survival rate zone	574	4.2E-02	7.9E-04
Functional loss	30 < ALSFRS	High survival rate zone	570	2.8E-02	1.0E-03

KL: Kullback-Leibler divergence

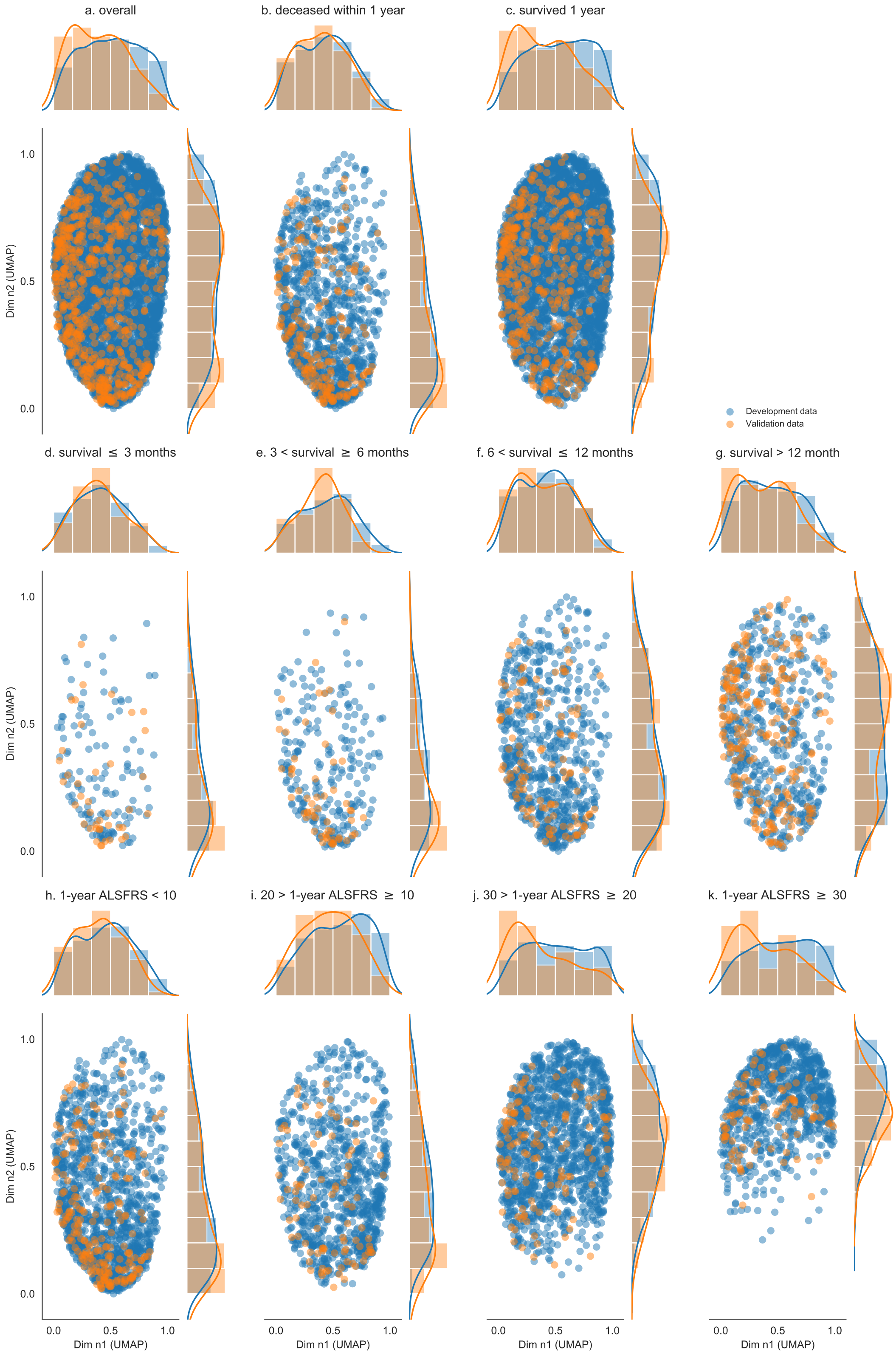

Figure 5. Distribution patterns for development and validation data (a). Distribution patterns for specific subsets of data based on the different outcomes. For 1-year survival, survivor and deceased patient populations are separated in respectively (b) and (c). For overall survival, patient population is divided based on survival range: less than 3 months (d), between 3 and 6 months (e), between 6 and 12 months (f) and above 12 months (g). For functional loss, patient population is divided based on ALSFRS range: less than 10 (h), between 10 and 20 (i), between 20 and 30 (j) and above 30 (k). Axes are dimensionless and come from UMAP dimension reduction.

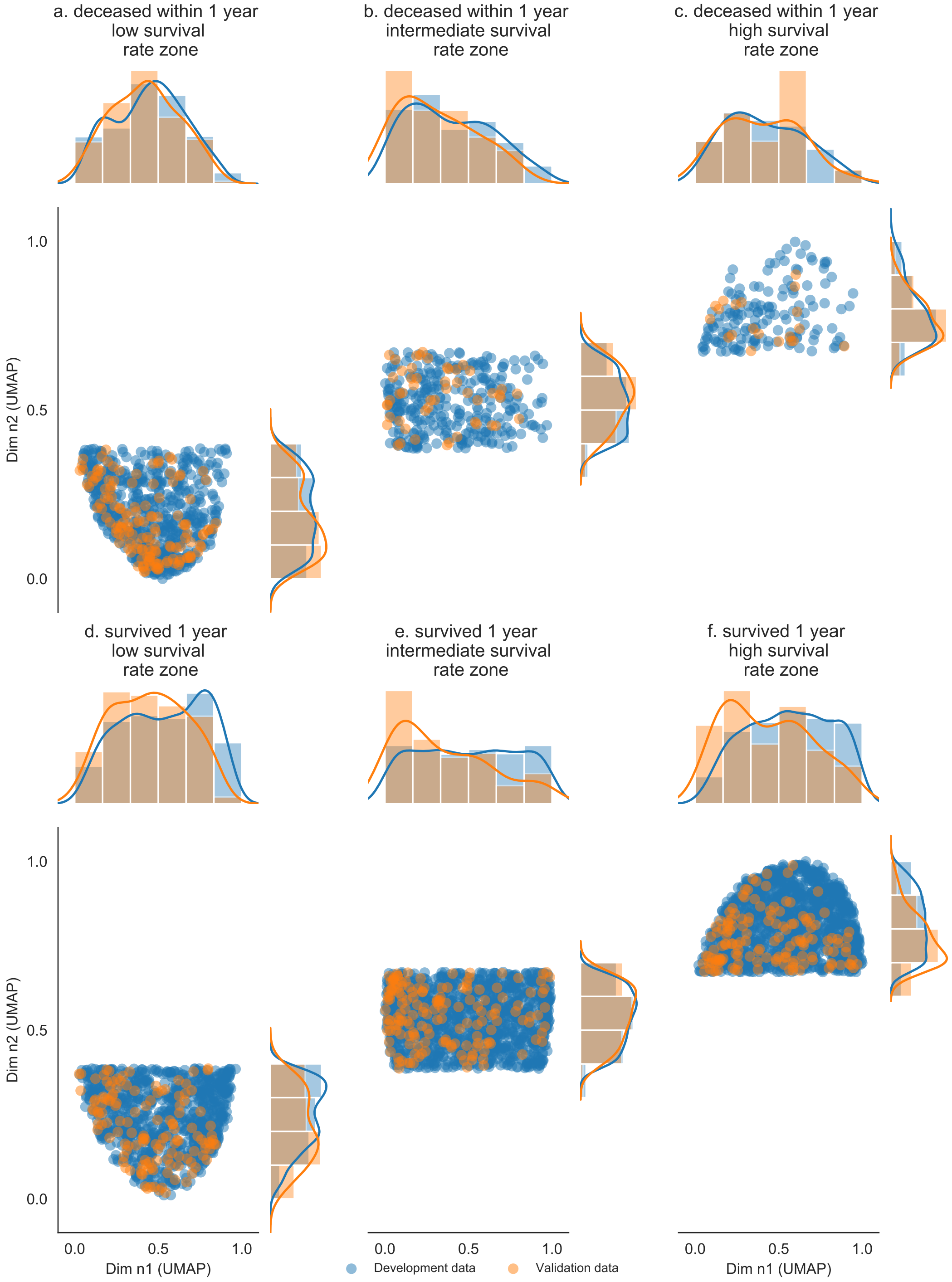

Figure 6. Distribution patterns for development and validation data for 1-year survival. Distribution patterns for specific subsets of data based on the survival outcome and the zones identified for prognosis estimation. Patients that are deceased within the first year are separated in 3 subsets based on zone membership in (a,b,c). Patients that survived are separated in 3 subsets based on zone membership in (d,e,f). Axes are dimensionless and come from UMAP dimension reduction.

Table 8. ANOVA testing on all 4 datasets (Trophos, Exonhit, PRO-ACT and real world).

Feature	F-statistic	p value	Result	Effect
Gender	7.10	9.36E-05	Independent (fail to reject H0)	0.00
Onset	7.27	7.36E-05	Independent (fail to reject H0)	0.00
Age	59.30	1.18E-37	Dependent (reject H0)	0.04
Symptom duration	23.72	3.10E-15	Dependent (reject H0)	0.01
Baseline weight	31.18	5.81E-20	Dependent (reject H0)	0.02
Baseline ALSFRS	119.34	1.35E-74	Dependent (reject H0)	0.07
Baseline est. ALSFRS rate	37.65	4.76E-24	Dependent (reject H0)	0.02

est.: estimated

Table 9. ANOVA testing on all 3 clinical trial datasets (Trophos, Exonhit, PRO-ACT).

Feature	F-statistic	p value	Result	Effect
Gender	0.39	0.30	Independent (fail to reject H0)	0.00
Onset	1.20	0.30	Independent (fail to reject H0)	0.00
Age	1.49	0.23	Independent (fail to reject H0)	0.00
Symptom duration	41.84	1.02E-18	Dependent (reject H0)	0.02
Baseline weight	22.70	1.57E-21	Dependent (reject H0)	0.01
Baseline ALSFRS	172.62	8.29E-73	Dependent (reject H0)	0.08
Baseline est. ALSFRS rate	17.48	2.75E-08	Dependent (reject H0)	0.01

est.: estimated

Table 10. Assumption testing before ANOVA testing.

Feature	Hom. (t-stat)	Hom. (p value)	Norm. (t-stat)	Norm. (p value)
Gender	7.10	9.36E-05	0.66	0
Onset	7.27	7.36E-05	0.56	0
Age	59.30	7.36E-05	0.99	3.04E-21
Symptom duration	41.56	1.64E-26	0.84	0
Baseline weight	16.32	1.50E-10	0.90	0
Baseline ALSFRS	46.26	1.79E-29	0.95	2.87E-37
Baseline est. ALSFRS rate	19.72	1.05E-12	0.76	0

Hom.: Homogeneity of variance **Norm.**: Normality

Additional information on projection space division

The division proposed in **Figure 3c** can be furthermore refined. The initial projection space in **Figure 7a** is divided into multiples small square cells as shown in **Figure 7b**. The less populated a cell, the wider the confidence interval associated with the survival probability within that cell and the less reliable the analysis of cell membership. The grid is used to identify survival patterns. These patterns help find a boundary for the two zones which maximises survival rate for the upper panel zone and minimises survival rate for the lower panel zone. Optimisation of the survival rate zone boundaries was performed by adjusting the previously straight-line boundaries to exclude patients with an uninformative outcome from high and low survival rate zones. Similarly to what was earlier presented, the UMAP projection space can be divided into three new zones with different survival rates: high, intermediate and low survival rate zones with respectively 90% (±4%), 77% (±4%) and 50% (±6%) as shown in **Figure 7c**. The low survival rate is slightly modified to −8% for the low survival rates zones. As for the simple division, the intermediate survival rate zone is non-informative as the zone's survival rate is similar to the overall patient survival rate of 79%.

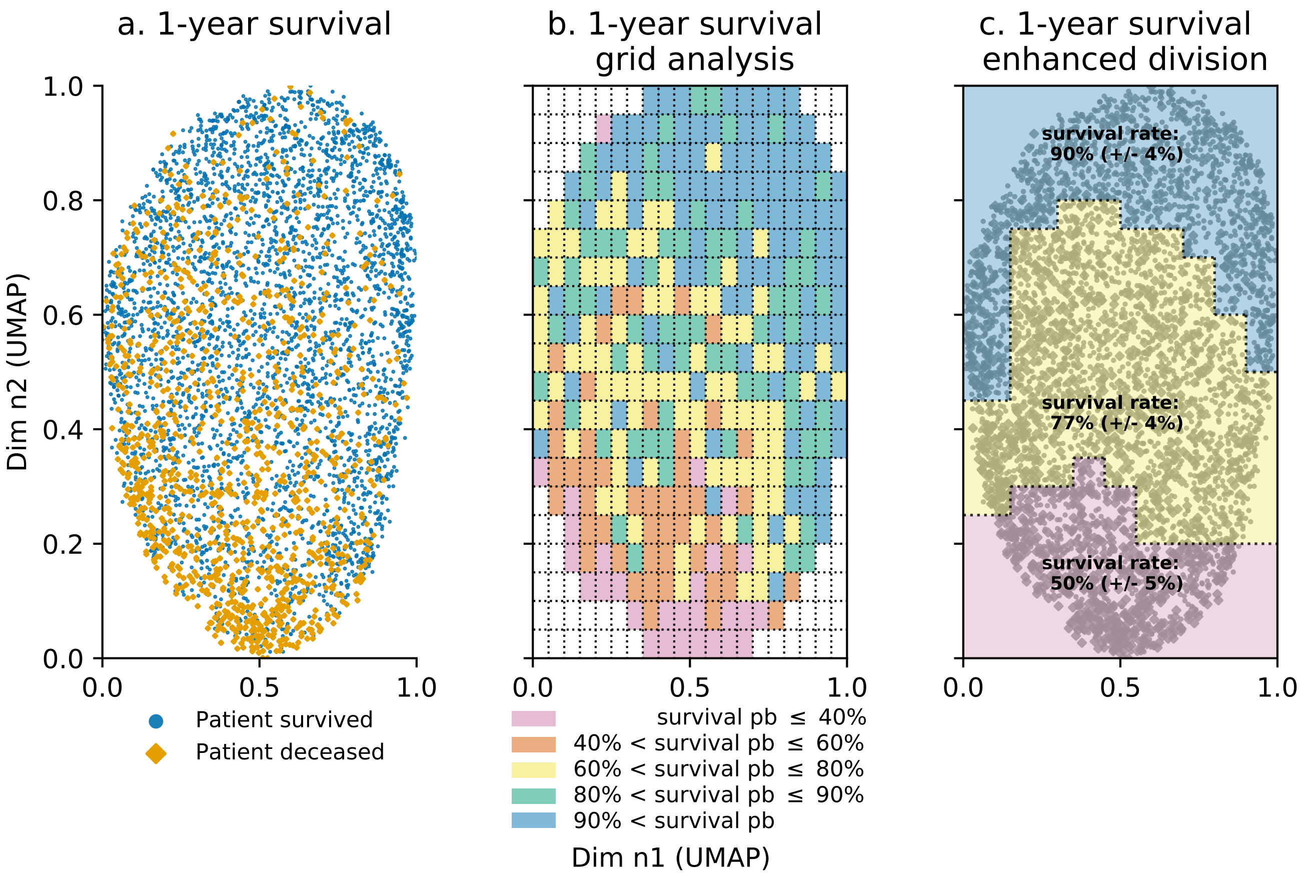

Figure 7. Enhanced 1-year survival projection space segmentation: initial 1-year survival distribution (a), projection space division using square cells and survival probability estimation per cell (b), resulting enhanced projection space division using cell survival probability distribution (c). Each point represents an individual patient. The projection space is divided in a square grid (b) with each cell having a specific survival rate computed based on patients belonging to that cell (which have either survived or deceased within the year). The overall space is divided in three zones based on the distribution pattern observed in square grid (c); the survival rate for each zone is calculated using patients belonging to each zone. Axes are dimensionless and come from UMAP dimension reduction.

References

1. Lenglet, T. *et al.* A phase ii- iii trial of olesoxime in subjects with amyotrophic lateral sclerosis. *Eur. journal neurology* **21**, 529–536 (2014).
2. Meininger, V. *et al.* Pentoxifylline in als: a double-blind, randomized, multicenter, placebo-controlled trial. *Neurology* **66**, 88–92 (2006).
3. Pro-act database. https://nctu.partners.org/ProACT/Home/Index. Accessed: 2020-01-01.
4. Pearson, K. Liii. on lines and planes of closest fit to systems of points in space. *The London, Edinburgh, Dublin Philos. Mag. J. Sci.* **2**, 559–572 (1901).
5. h. Taguchi, Y., Iwadate, M. & Umeyama, H. Heuristic principal component analysis-based unsupervised feature extraction and its application to gene expression analysis of amyotrophic lateral sclerosis data sets. In *2015 IEEE Conference on Computational Intelligence in Bioinformatics and Computational Biology (CIBCB)*, DOI: 10.1109/cibcb.2015.7300274 (IEEE, 2015).
6. Tang, M. *et al.* Model-based and model-free techniques for amyotrophic lateral sclerosis diagnostic prediction and patient clustering. *Neuroinformatics* **17**, 407–421 (2019).
7. Maaten, L. v. d. & Hinton, G. Visualizing data using t-sne. *J. machine learning research* **9**, 2579–2605 (2008).

8. Schubert, E. & Gertz, M. Intrinsic t-stochastic neighbor embedding for visualization and outlier detection. In *International Conference on Similarity Search and Applications*, 188–203 (Springer, 2017).

9. McInnes, L., Healy, J. & Melville, J. Umap: Uniform manifold approximation and projection for dimension reduction. *arXiv preprint arXiv:1802.03426* (2018).

10. Chiò, A. *et al.* Als clinical trials: do enrolled patients accurately represent the als population? *Neurology* **77**, 1432–1437 (2011).
